# Experiences, perceptions and expectations of health services amongst marginalized populations in urban Australia: A meta‐ethnographic review of the literature

**DOI:** 10.1111/hex.13386

**Published:** 2021-12-14

**Authors:** Kirsten Baker, Jon Adams, Amie Steel

**Affiliations:** ^1^ Faculty of Health, School of Public Health Sydney University of Technology Sydney Australia

**Keywords:** delivery of health care, health equity, health services, public health, social determinants of health, vulnerable populations

## Abstract

**Background:**

Poor health outcomes amongst marginalized groups result in part from health inequities related to social and structural determinants of health. Marginalized people report higher incidences of comorbidities, chronic disease and adverse health behaviours than their nondisadvantaged peers. The objective of this review is to examine marginalized Australians' experiences of and access to community‐based primary health services in urban locations.

**Methods:**

A systematic search incorporating related MeSH terms and synonyms pertaining to marginalized Australian populations and their health‐seeking was conducted across seven databases. We included qualitative studies that reported experiences of health‐seeking within community‐based primary health care in metropolitan Australia. Participant populations experiencing marginalization due to social stigma and isolation, early‐life disadvantage, poor health and/or financial hardship were included. A meta‐ethnographic framework was used to synthesize themes across selected studies and researcher triangulation was employed to develop higher‐order themes.

**Results:**

Search results revealed 26 studies included for critical appraisal and synthesis. Seven higher‐order themes were developed describing experiences of health service engagement amongst marginalized groups: (1) Understanding the patient within the context of family and community, (2) Health and cultural beliefs influence health‐seeking, (3) Lack of information and poor cultural competence limit utilization of services, (4) Motivation for treatment influences health service engagement, (5) Accessing services, a spectrum of experience—from discrimination to validation, (6) Navigating a complex system in a complex society, (7) Preferences for health care and expectations for systemic change.

**Conclusion:**

Marginalized Australians experience health disadvantage across micro, meso and macro levels of health system navigation and commonalities in health‐seeking were identified across each of the distinct marginalized groups in our analysis. This review outlines important areas of consideration for health care provision and policy development essential to helping address health inequities for a diversity of marginalized populations.

**Patient or Public Contribution:**

Whilst patient voices were reported across all studies included within this review, no further patient or public contribution applies to this study.

## 1 INTRODUCTION

Health inequality and subsequent disparities that result from inequitable access to essential health services are recognized as important social and public health challenges.^1^ Disadvantaged populations—such as asylum seekers, refugees, indigenous peoples, ethnic or sexual minorities and the homeless—face marginalization in a complex and sometimes punitive health care system,^2^, ^3^, ^4^ contributing to consistently poor health outcomes compared to their nondisadvantaged peers.^2^, ^3^ Marginalized people, defined as ‘populations outside of mainstream society’^5^ are frequently stigmatized in their health‐seeking and are reported to commonly receive poor quality or inappropriate health care that fails to adequately meet their needs.^2^, ^3^, ^6^ Globally, evidence of a significant increase in the awareness of social determinants and their relationship to health has been reported in medical research over the past 10 years.^4^

In Australia, the monitoring of social determinants and the distribution of health across social groups has been prioritized at the policy level.^7^ For many Australians, complex health needs, cultural and socioeconomic factors or geographical location may affect their ability to access health services.^7^ Furthermore, people living in the lowest socioeconomic or rural/remote areas, those with a disability and those who identify as Aboriginal and Torres Strait Islander, experience higher rates of illness, hospitalization and morbidity than other Australians.^7^ Health policies focussed on ‘closing the gap’ between indigenous and nonindigenous health outcomes have been unable to meet targets while chronic disease burdens and multimorbidities continue to be recorded at disproportionate levels.^2^, ^7^ Similarly, growing numbers of homeless and ‘at risk’ youth signify increasing intergenerational disadvantage and poverty^3^, ^6^ while recent census data recorded 58% of Australians experiencing homelessness were below 35 years of age.^7^ Population health analysis shows Australians from low socioeconomic backgrounds fare worse concerning health risk factors and chronic health conditions, such as diabetes, cancer, stroke and anxiety.^7^ Low health literacy places an unfair burden on racial and ethnic minority, CALD and refugee and asylum seeker populations. Consequently, these groups risk poor health outcomes and adverse health behaviours due to misunderstanding, which results in decreased engagement with services,^7^ further contributing to poor health status and subsequent health inequalities.^7^

Primary health care (PHC) constitutes the frontline of Australia's health care system, delivering services outside a hospital environment, often in a community setting without the need for referrals. These services, funded through the Medicare Benefits Scheme additional to government, nongovernment, private and philanthropic sources, are often the main or sole contact point between the marginalized and formal health care provision. Access to PHC services has been confirmed as improving population health outcomes and reducing inequality^7^ highlighting the important role of PHC professionals in addressing the health care needs of marginalized groups. However, Australia trails behind other high‐income nations in training and preparing all health professionals to provide culturally responsive health care.^2^ Concepts of cultural competency and culturally responsive communication have been outlined as essential skills for health care providers to reduce health disparities.^2^, ^8^ Culturally responsive communication, a cornerstone of person‐centred care, is defined as ‘communicating with awareness and knowledge of cultural differences and attempting to accommodate those differences’ and is inclusive of linguistic, cultural, racial/ethnic, religious and sexual diversity.^8^ Understanding the experiences of marginalized groups in their health‐seeking is imperative for health workers to be able to provide equitable and best‐practice care for those disadvantaged by the existing health system. Encouraging further consideration of the racial, cultural, sexual, linguistic and religious diversity of health care users helps promote quality person‐centred care that helps meet the complex needs of the marginalized.^9^

Previous international research has outlined the need to address interventions for marginalized and excluded populations as a whole, rather than focussing on subpopulations defined by singular risk factors and needs.^3^ Literature reviews detailing extensive research into the health care experiences of various subpopulations of marginalized Australians^10^, ^11^ highlight a gap in enquiry with particular reference to health care experiences *across* marginalized populations as a whole. In response, the following review seeks to fill this study gap and address a ‘defined need’ by using a wider lens to explore themes of health care experience *across* these populations.

## 2 METHODS

### 2.1 Aim and objectives

This meta‐ethnography aims to explore the health care experiences of diverse groups of marginalized populations within Australia. Specifically, this study seeks to understand what marginalized groups are reporting about their experiences of and access to urban PHC. Through the collection, collating and appraisal of reported experience, this review aims to provide a useful interpretation of health service engagement *across* marginalized Australian populations.

### 2.2 Methodology

Meta‐ethnography, well established in health policy and strategy research,^12^ provided the methodological framework for this enquiry to assess and collate consumer experiences of health service provision and, most importantly, give ‘voice’ to these experiences.^12^, ^13^ The philosophical underpinnings and approach to this ethnographical research are drawn from an interpretivist paradigm^13^ and reference activist and candidacy frameworks in advocating for the marginalized. Guidance in the reporting of this review was derived from the eMERGe guidelines,^12^ which aim to help inform the improvement of service‐user outcomes within the health field.^12^ We followed the seven‐phase process for conducting a meta‐ethnography conceptualized by Noblit and Hare^13^ and elucidated upon it in recent literature.^14^ Meta‐ethnography diverges from other forms of qualitative synthesis by reinterpreting conceptual (primary) themes from individual studies and translating and synthesizing these studies through the development of higher‐order themes.^13^, ^14^ A description of our application of this process is detailed in Table 1.

**Table 1:** Application of the seven phases for meta‐ethnography

Seven phases for conducting a meta‐ethnography conceptualized and outlined by Noblit and Hare	Description of each phase as a process	Methodological checklist and process conducted in each phase of this review	Outcome of each of the seven phases conducted for this review
*Phase 1: getting started*	Identifying an area of interest, determining if qualitative synthesis is best suited to inquiry	Research team agrees that a qualitative synthesis of knowledge concerning the topic of interest is applicable and agrees that investigating this phenomenon will be of value in health care delivery and policy	Research question is identified: What are the experiences, perceptions and expectations of health services amongst marginalized populations in urban Australia?
*Phase 2: deciding what is relevant to the initial interest*	a. Define the focus of the synthesis b. Locate relevant studies c. Decisions to include studies d. Quality appraisal	a. We chose to focus on qualitative research that reported the experiences of diverse marginalized participant populations and limited study location to metropolitan areas so as to be able to focus our enquiry b. We conducted a systematic search across seven databases with limiters placed on peer‐reviewed publications and studies reported in English c. A thorough assessment of the methods section of each paper was conducted by K. B. and checked by A. S., to determine study inclusion d. The CASP quality appraisal tool was applied (by K. B.) to help assess the rigour and quality of each study	a. The team discussed the parameters of our search process and outlined inclusion/exclusion criteria b. The search process yielded 2256 results, a flowchart outlining the search process was produced c. Additional studies were excluded in the final screening phase, 26 studies were revealed for inclusion in this review d. The quality of the studies included in the review varied though all were deemed to present data that could provide a worthwhile contribution to our topic of enquiry
*Phase 3: reading the studies*	Reading and rereading included studies; extracting data	All studies were printed in full, read and reread and hand‐annotated by K. B.	Data extraction form was developed and completed by K. B. then reviewed and amended (where applicable) by A. S. and J. A.
*Phase 4: determining how the studies are related*	Rereading studies and outlining key concepts and themes	Studies were uploaded into NVivo qualitative software. Studies were initially grouped according to their participant population. Participant quotes and key first‐author observations were highlighted in the text	A list of key concepts and descriptors for each study was developed by K. B. and then reduced into categories in consultation with A. S. and J. A.
*Phase 5: translating the studies into one another*	Comparing and contrasting metaphors across studies	Descriptions and metaphors were compared across each of the included studies akin to a constant comparison process as clearer conceptual themes began to emerge	Translation tables were produced in NVivo during this iterative phase by K. B. and shared with J. A. and A. S. Discussion about emerging and repetitive themes and how these were to be coded ensued
*Phase 6: synthesizing the translations*	Bringing studies together—the whole (synthesis) bigger than the individual parts	Key concepts and descriptors of each study were compared and contrasted to determine how they related. Distinct themes emerged that indicated the reciprocal nature of the synthesis	Higher‐order themes were conceptualized and outlined and a final line of argument synthesis outcome was able to be defined: the presence of seven key commonalities or themes influenced the health‐seeking of diverse groups of marginalized Australians
*Phase 7: expressing the synthesis*	Presenting the findings of the synthesis	K. B. drafted the manuscript (in line with the eMERGE guidelines) and developed diagrams to supplement the process of conducting the meta‐ethnography with review and input from J. A. and A. S.	Research findings are composed in narrative and diagrammatic form with a summary of findings and outlined strengths and limitations. Completed article submitted for peer‐review publication

### 2.3 Search strategy

The search strategy for this meta‐ethnography incorporated the following four themes: health services, vulnerable or disadvantaged populations, experiences or perceptions and Australian‐based studies. Synonyms and MeSH terms relevant to the four themes were applied as shown in Table 2. Databases searched by K. B. included Informit, Medline, Scopus, PsychInfo, Embase, AMED and CINAHL, details can be found in the Supporting Information (Appendix S1). Searches were limited to peer‐reviewed scholarly articles and papers reported in the English language.

**Table 2:** Medline database search strategy

	Medline (OVID) search string terms with results
1	Health behavior (48847)
2	(experience* Perception*).mo. or Preference*.ti.ab. (1532118)
3	((marginalised or vulnerable or at risk or underserved or disadvantaged or minority) adj3 (population* or communit* or group*)). ti. ab.
4	(experience* or Perception*). mp. or Preference*.ti.ab. (1532118)
5	Health Knowledge, Attitudes, Practice/(108552)
6	Attitude to Health (82770)
7	Patient Satisfaction/(80235)
8	Patient Preference/(8042)
9	‘Patient Acceptance of Health Care’/(45238)
10	Exp Health Services/(2094699)
11	Health care utilisation.mp. (581)
12	Health care.mp. (774999)
13	Health provision.mp. (390)
14	Australia. ti,ab. (90228)
15	10 or 11or 12 or 13 (2572487)
16	1 or 2 or 5 or 6 or 7 or 8 or 9 (1774591)
17	3 and 14 and 15 and 16 (178)
18	Australia/(100780)
19	3 and 15 and 16 and 18 (142)
20	Vulnerable Populations/(10049)
21	3 or 20 (61690)
22	14 or 16 (153442)
23	15 and 16 and 21 and 22 (287)

### 2.4 Eligibility criteria

**Figure 1:**
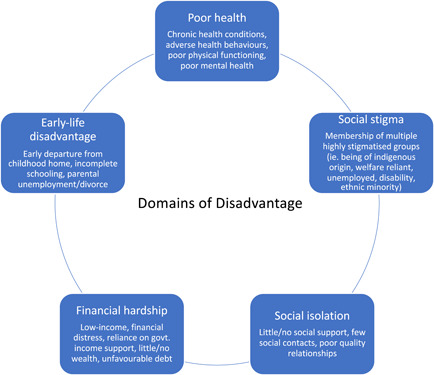
The five Domains of Disadvantage defining inclusion criteria for this review

Papers were included if they: (1) reported new empirical data; (2) focused on the experiences and perceptions of marginalized patients/communities regarding their perspectives of community‐based urban PHC; (3) were collected in Australian settings; (4) the sample population qualified for at least two of the five listed Domains of Disadvantage identified by previous authors^15^ chosen to define marginalization for the purposes of this review (refer to Figure 1).^15^

We excluded home‐delivered care or health services accessed within prison environments or other institutions, such as detention centres, aged or other care facilities and schools as well as papers reporting on paediatric, palliative, geriatric, emergency, midwifery and antinatal care. Additionally, we excluded health promotion interventions targeted at marginalized groups within the broader community as well as within school environments.

### 2.5 Screening and selection

An initial pool of 2256 citations was retrieved for systematic refinement and review and uploaded into reference‐management software (Endnote X9). Titles were screened against eligibility criteria by K. B. Seventy‐five original research articles reporting on marginalized populations met our inclusion criteria. Of these, the 32 quantitative papers were excluded and upon in‐depth reading of the remaining studies, K. B., A. S. and J. A. agreed to exclude 14 papers that did not meet the inclusion criteria for this study. The search process for this meta‐ethnography concluded with a review and synthesis of 26 qualitative papers.

### 2.6 Data extraction

All studies included in the synthesis were printed in full and hand‐annotated during the critical reading phase by K. B. These same files were uploaded to NVivo software with Stage 1 descriptive coding—summarizing the topic of textual passage—performed independently by K. B. and shared with A. S. and J. A. We aimed to triangulate the second‐stage coding process to ensure the accuracy of coding and consistency of theme development. Each researcher identified and recorded concepts themes and metaphors from each full primary study. Data were extrapolated in the form of the authors' concepts and themes from primary studies as well as quotes (in vivo coding) from study participants. The Stage 2 coding process identified final overarching concepts and themes. Characteristics of included studies are outlined in Table 3. The CASP checklist was used for the assessment of quality amongst included studies.^16^, ^17^ It indicated that all papers included for synthesis were of value to the research enquiry despite some discrepancies in strengths and weaknesses pertaining to the quality of reporting or methodological clarity. The critical appraisal was conducted by one reviewer (K. B.) and checked by A. S. and is detailed in Table 4.

**Table 3:** Summary details on included articles

ID	Author (year)	Aims	Participants	Methodology	Themes reported in article
1	Abbott et al. (2016)	To examine expectations & experiences of GP care of women leaving prison	Women (*n* = 29) interviewed pre & post and 1–6 months postrelease from prison	Interviews with thematic analysis and the candidacy framework	1. Needs & vulnerability postrelease 2. Postrelease continuity of care 3. Expectations & experiences of GP services
2	Abbott et al. (2017)	Medical homelessness and candidacy: women transiting between prison and community care	29 women interviewed postrelease from prison	Interviews with thematic analysis and candidacy theoretical framework	1. Prison as an opportunity to improve health 2. Stigma in and out of prison 3. Feeling let down by health care providers
3	Cuesta‐Briand et al. (2014)	Explores experiences of diabetes care & inequities in clinical outcomes for the disadvantaged	Indigenous and nonindigenous groups socioeconomically disadvantaged populations.	Focus groups + in‐depth interviews interpretive influenced by a critical paradigm using design of conflict theory	1. Preferences for Aboriginal Controlled Community Health Organisations (ACCHO) 2. Perceptions of need for health care services varied greatly 3. Previous negative health care seeking experiences 4. Lack of information & awareness of key services of potential benefit 5. Cost as a barrier to service access
4	Freeman et al. (2014)	Study experiences of primary health services amongst disadvantaged groups	65 indigenous & nonindigenous participants from disadvantaged backgrounds	Community capacity workshops (Labonte and Laverack)	1. Inclusivity 2. Empowerment
5	Pallotta‐Chiarolli and Martin (2009)	To explore mental health of bisexual‐ identifying & behaving adolescents & young people	30 adolescent/young people (15–25 years) identifying as bisexual	Semistructured interviews	1. Problematic representation 2. Discrimination 3. Access & use of health services
6	Black et al. (2018)	To identify what factors hinder or enable referrals between social & health care for homeless youth with mental health problems	10 homeless youth	in‐depth interviews; phenomenological design	1. Referral barriers—lack of follow‐up, abrupt discharge, ‘treated like a number’ 2. Referral facilitators—support, advocacy, client‐centred care
7	Darbyshire et al. (2006)	Describe the experiences of young homeless people and their engagement with health and social care services	10 people (7 female, 3 male) aged between 16 & 24 years who identified as homeless with experience of mental health problems	Semistructured interviews, theory from childhood youth studies	1. Labelling 2. Hasty assessment 3. Lack of explanations 4. Lack of personal control 5. Poor service co‐ordination 6. Trust & respect as cornerstones of a therapeutic relationship 7. Feeling heard
8	Robards et al. (2019)	To understand how marginalized youth navigate the health system and the role of technology in this navigation	41 young people (mean age 19 years) belonging to one or more marginalized groups: rural/remote dwelling; homeless; Aboriginal; sexuality/gender diverse; refugee background—with a chronic/complex health condition and/or disability	Qualitative arm of the larger mixed‐methods project: 4 interviews over a 14‐month period	1. Technology provides opportunities for service engagement 2. Convenience, engagement, affordability & perceived effectiveness 3. Discrimination leading to poor care 4. Multiple marginalization provides greater challenges 5. Acknowledged health system fragmentation mitigated by navigation support
9	Jiwrajka et al. (2017)	A focus on cultural and language barriers in the delivery of health care	A Burmese refugee woman who had fled Ethno‐religious persecution with a recent diagnosis of type 2 diabetes	Case report with a qualitative component	1. Confusion about diagnosis 2. Dangers of miscommunication for health outcomes
10	McBride et al. (2017)	Description of a refugee health service and clients' experiences	18 refugees (16 male, 2 female)	Mixed methods: semistructured interviews	1. Appreciation for access to health care 2. Recognized benefit of integrated care 3. Experiences of culturally competent care
11	Metusela et al. (2017)	Examining experiences of sexual & reproductive health of migrant & refugee women from diverse cultural & religious backgrounds	169 migrant & refugee women (mean age 35 years)	In‐depth interviews, focus groups utilizing social constructivist epistemology employing thematic analysis	1. Lack of knowledge about sexual/reproductive health & screening 2. Cultural barriers to accessing sexual/reproductive health knowledge 3. Negative sexual/reproductive health outcomes
12	McMichael and Gifford (2010)	Reports on sexual health literacy amongst recently arrived refugee youth	142 refugees between 16 and 24 years old (67 males, 75 females)	Focus group discussions & in‐depth interviews	1. Inadequate information on STI's & HIV 2. Taking risks, taking care: contraception, abstinence, trusting sexual relationships, avoiding risky ‘types’
13	Gholizadeh et al. (2011)	To understand the lack of service utilization in cardiovascular health amongst middle eastern women	66 participants mean age 44 years	Focus groups; constructionism, interpretivism & grounded theory	1. Perceptions of CVD risk‐fatalism 2. Stress & acculturation 3. Cultural barriers to risk‐reducing behaviours
14	Rose and Harris (2015)	Explore the levels of self‐management support from GP's towards ethnically diverse groups with diabetes	28 diabetes patients, Arabic speaking (*n* = 11), Vietnamese speaking (*n* = 8), English speaking (*n* = 9)	Semistructured interviews phenomenological analysis	1. Experiences of GP information quality 2. Experiences of GP consultation style
15	Maneze et al. (2018)	Explore the way Filipino migrants experience the management of their chronic health conditions within the context of living in a host country	58 Filipino‐Australian migrants; mean age 67 years; 88% female; chronic diseases: hypertension (91%), heart disease, diabetes, comorbidities in 49% of participants	Qualitative components of a larger mixed‐methods study: focus group, exploratory sequential design with thematic analysis	1. Perceptions of abundance scarcity within an Australian health care system 2. Juggling self‐care responsibilities within the Australian lifestyle
16	Prasad‐Ildes and Ramirez (2006)	To consult with CALD consumers & glean their perceptions of mental illness prevention	28 participants from Arabic & Spanish speaking backgrounds as well as participants from Bosnia, Iran and Filipinas with experiences of mental illness	Bespoke instrument design—consumer consultation	1. Informed mental illness awareness support for community & religious leaders 2. Acculturation issues—language, lifestyle 3. Intergenerational differences—support for children
17	Wynaden et al. (2005)	To understand the values & beliefs of people from Asian communities about mental illness	10 participants (3 women, 7 men) were recruited by convenience sampling, the majority were health care workers and members of the study community	Semistructured interviews content analysis	1. Shame & stigma 2. Causes of mental illness 3. Family reputation 4. Hiding up 5. Seeking help 6. Lack of collaboration
18	Körner (2007)	Reporting on circumstances of late HIV diagnosis and perceptions of HIV risk for people from diverse cultural backgrounds	29 participants were recruited from a multicultural HIV/AIDS and Hep C health service. 22 men, 7 women.	Semistructured interviews; grounded theory—iterative analysis	1. Circumstances of diagnosis 2. Meaning of HIV diagnosis 3. Perceptions of risk
19	Vatcharavongvan et al. (2014)	To explore the health needs of both Thai women and men, including physical, mental, familial & social aspects of health	Purposefully sampled participants (*n* = 21), 17 women & 3 men. Mean age 53 years	Gender‐segregated focus groups; thematic content analysis	1. Positive experiences in Australia 2. Physical health problems 3. Mental health problems 4. Familial & social problems
20	Ho and Maher (2008)	To explore the influence of cultural beliefs and practices on the vulnerability to blood‐borne viral infections	58 participants were recruited through a mix of snowball & theoretical sampling; 86% male, mean age 31 years	In‐depth interviews, observation & fieldnotes	1. Health beliefs 2. Cultural characteristics 3. Knowledge & environmental constraints 4. Structural & institutional factors
21	Coupland et al. (2009)	Explore barriers to HCV treatment uptake and inform policy & practice that promote treatment amongst Indo‐Chinese IDU's	23 participants from Cambodia (*n* = 5), Laos (*n* = 8) & Vietnam (*n* = 10). 6 female, 17 male	Fieldwork observation, in‐depth interview, inductive analysis, constructivist theoretical framework, grounded theory	1. Barriers to HCV treatment uptake 2. A ‘one‐off’ chance for subsidized treatment 3. Uncertainty about the capacity to complete treatment: lack of support & resources 4. Reluctance to access the health system 5. Institutional barriers to accessing treatment
22	Kendall and Barnett (2015)	Understanding the factors that influence the participation of Aboriginal people in their use of mainstream health services	34 Aboriginal participants (the total number for the study was 39, including nominated health workers. The participant population were from rural, regional & metropolitan areas (*n* = 12)	Community‐based participatory approach—focus groups, interviews. Constructivist grounded theory, constant comparison	1. Historical reluctance to engage with the medical system 2. Cultural incompetence of the system 3. Colonial communication styles 4. Clash with the collective approach to disease 5. Health being more than a medical state
23	Thompson et al. (2009)	To explore and describe the role that alcohol plays in the lives of HIV + Aboriginal people in W.A.	20 Aboriginal participants (4 males, 16 females) mean age 33 years. Participants are both rural (*n* = 6) & metropolitan (*n* = 14) residing	Semistructured interviews, thematic analysis	1. ‘Good times’: the days before HIV 2. ‘Too drunk and stupid’: alcohol as a risk for acquiring HIV 3. ‘The most biggest shock of my life’: diagnosis during treatment for alcohol misuse 4. ‘Escape but it still knocks at your door’: coping with the diagnosis 5. ‘Only after I got drunk’: helping with disclosure 6. ‘I slowed right down’: changing lifestyle 7. Alcohol interfering/coping with medication; as a social activity
24	Treloar et al. (2016)	To add to the research on HCV treatment and policy development with the aim of contributing programmes relevant to Aboriginal people	39 Aboriginal people with HCV, 15 female, 1 transgender; mean age 40 years Recruitment via ACCHS, hospital clinics treating HCV, needle and syringe programmes, drug & alcohol services, community health & social services	Telephone interview, thematic analysis	1. Lack of information, lack of support 2. Valuing health 3. Concerns about side effects 4. Treatment experiences: the anticipation of difficulties vs. positive experience & ACCHS
25	Wallace et al. (2018)	Explore patient experiences of HCV treatment & cure and explore barriers to treatment uptake amongst Aboriginals	18 people with HCV who had accessed treatment through the Victorian Aboriginal Health Service—9 males, 9 females	Semistructured interviews	1. Context of HCV within a community: shame around the transmission 2. Context of the liver clinic within VAHS: patient‐centred, safe, not medicalized 3. Being treated: direct health benefits
26	Dimer et al. (2013)	Evaluation of the implementation of a culturally appropriate cardiac rehab model assessing uptake, lifestyle risk factors	48 Aboriginal participants at an Aboriginal Medical Service (AMS) for cardiac rehabilitation—64% female	Mixed methods: interviews and ‘yarning’ sessions	1. Overwhelming preference for interventions delivered within AMS 2. Community support for exercise and health education as prevention

**Table 4:** Critical appraisal of included articles

Checklist item	Author and year																									
	Abbott et al. (2017)	Abbott et al. (2016)	Black et al. (2018)	Coupland et al. (2009)	Cuesta‐Briand et al. (2014)	Darbyshire et al. (2006)	Dimer et al. (2012)	Freeman et al. (2014)	Gholizadeh et al. (2011)	Ho and Maher (2008)	Jiwrajka et al. (2017)	Kendall and Barnett (2015)	Körner 2007	Maneze et al. (2017)	McBride et al. (2017)	McMichael and Gifford (2010)	Metusela et al. (2017)	Pallotta‐Chiarolli and Martin (2009)	Prasad‐Ildes and Ramirez (2006)	Robards et al. (2019)	Rose and Harris (2015)	Thompson et al. (2008)	Treloar et al. (2016)	Wallace et al. (2018)	Wynaden et al. (2005)	Vatcharavongvan et al. (2014)
Was there a clear statement of the aims of the research?	Y	Y	Y	Y	Y	Y	Y	Y	Y	Y	Y	Y	Y	Y	Y	Y	Y	Y	Y	Y	Y	Y	Y	Y	Y	Y
Is a qualitative methodology appropriate?	Y	Y	Y	Y	Y	Y	Y	Y	Y	Y	Y	Y	Y	Y	Y	Y	Y	Y	Y	Y	Y	Y	Y	Y	Y	Y
Was the research design appropriate to address the aims of the research?	Y	Y	Y	Y	Y	Y	Y	Y	Y	Y	Y	Y	Y	Y	Y	?	Y	?	Y	Y	Y	Y	Y	Y	Y	Y
Was the recruitment strategy appropriate to the aims of the research?	Y	Y	Y	Y	Y	Y	Y	Y	Y	Y	Y	Y	Y	Y	Y	Y	Y	Y	Y	Y	Y	Y	Y	Y	Y	Y
Was the data collected in a way that addressed the research issue?	Y	Y	Y	Y	Y	Y	Y	Y	Y	Y	?	Y	Y	Y	Y	Y	Y	Y	Y	Y	Y	Y	Y	Y	Y	Y
Has the relationship between researcher and participants been adequately considered?	Y	Y	Y	Y	Y	Y	?	Y	Y	Y	Y	Y	Y	Y	?	Y	Y	Y	?	Y	Y	Y	Y	Y	Y	Y
Have ethical issues been taken into consideration?	Y	Y	Y	Y	Y	Y	Y	Y	Y	Y	Y	Y	Y	Y	Y	Y	Y	?	N	Y	Y	Y	Y	Y	Y	Y
Was the data analysis sufficiently rigorous?	Y	Y	Y	Y	Y	Y	Y	Y	Y	Y	Y	Y	Y	Y	Y	Y	Y	Y	?	Y	Y	Y	Y	Y	Y	Y
Is there a clear statement of findings?	Y	Y	Y	Y	Y	Y	Y	Y	Y	Y	Y	Y	Y	Y	Y	Y	Y	N	Y	Y	Y	Y	Y	Y	Y	Y
How valuable is the research?	V	V	V	V	V	V	V	V	V	V	V	V	V	V	V	V	V	V	V	V	V	V	V	V	V	V

Abbreviations: N, no; NV, not valuable; V, valuable; Y, yes;  ?, cannot tell.

### 2.7 Data analysis

The participant characteristics of the primary studies were compared by K. B. to determine how they related. The ethnography aimed to draw inferences from the collective experiences of health care for participants across different marginalized groups. France et al. outline the importance of refutational and reciprocal transition when conducting translation.^12^ It is important to understand which studies support or reciprocate the others' findings and equally, if there are studies included in the final pool that may refute these findings. The final pool of studies (*n* = 26) each fell within one or more of six distinct participant groups that met the inclusion criteria for included marginalized populations within this review: indigenous Australians; CALD populations; refugee and asylum seekers; socioeconomically disadvantaged or homeless populations; disadvantaged youth.

## 3 FINDINGS

**Figure 2:**
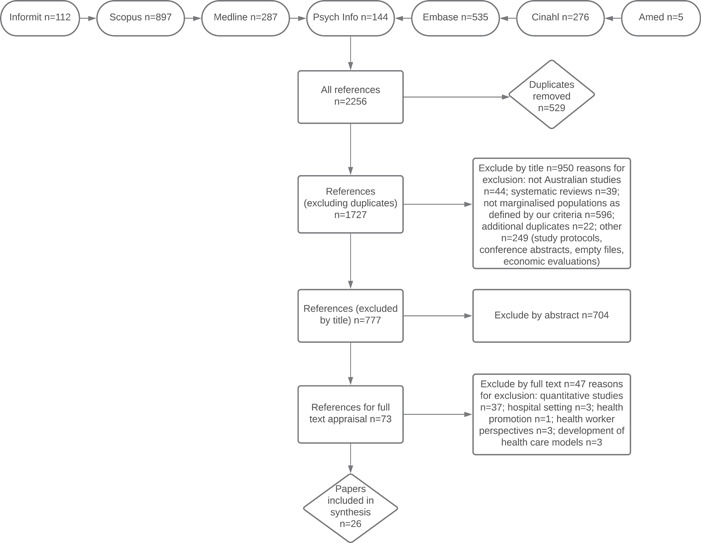
Flow chart outlining search process

**Figure 3:**
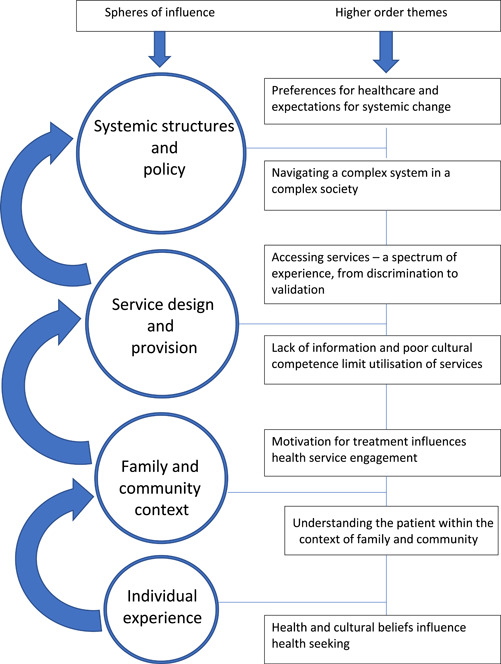
Framework outlining spheres of influence and related conceptual themes of health service engagement amongst marginalized Australians

The 26 original research articles included in this review (search process results are outlined in Figure 2) incorporate a variety of study designs and their findings give insight into the experiences and perceptions of health service use amongst a representative cross‐section of marginalized groups within Australia. Seven main themes were revealed in our analysis and echoed *across all six* of the diverse participant populations included in this synthesis, highlighting consistent reciprocal transition in the translation process—where each study supports in part or whole the findings of the other studies within the pool.^12^ Together these seven themes (collated in Table 5) tell a story of the contemporary health‐seeking experience of marginalized Australians. This new interpretation or conceptual ‘framework’—known as a *line of argument synthesis*—was developed in the final stage of analysis.^13^, ^14^ A line of argument infers that tertiary level analysis and synthesis across studies builds a more generalizable theoretical understanding, and an interpretation of the relationship between themes, where the whole becomes greater than the sum of its individual parts.^13^, ^14^, ^18^, ^19^ Our interpretation signals a collective experience of health service engagement for marginalized Australians, where the seven themes can be mapped with some overlap across different levels of influence, from the individual or community perspective through to an appraisal of the characteristics of service provision, and finally in reflection of systemic structures, governance and policy. This interpretation is presented in diagrammatic form^14^ in Figure 3. To aid clarity, each included study outlined in the below text is referred to by a number (as allocated in Table 3).

**Table 5:** Higher‐order themes, subthemes and descriptors

Higher‐order theme	First author ID^a^	Subthemes	First author ID	Description
Understanding the patient within the context of family and community	1, 2, 5, 8, 11, 12, 13, 15, 16, 17, 18, 19, 20, 21, 22, 23, 24, 25, 26			Describes how familial relationships and community support impact the way in which participants viewed their health options. Concept of a family as both a motivation for and source of shame pertaining to health‐seeking is discussed. Multidimensional and complex roles of intergenerational duty within the family, community and across cultures inform health‐seeking
Health and cultural beliefs influence health‐seeking	9, 12, 13, 15, 17, 18, 19, 20, 22, 23	‐Perceptions of risk ‐Cultural expectations related to gender, indications of female disadvantage	5, 11, 12, 13, 15, 17, 18, 19, 20, 22, 23, 24, 25 8, 11, 12, 13, 17, 18, 19	Highlights the effects of embedded belief systems, including risk‐taking, fatalistic attitudes and therapeutic preferences for traditional medicines, on the participants' experiences of health care. The role of gender in relation to cultural expectations and how this influences engagement with health services, often perpetuating disadvantage, is unpacked
Lack of information and poor cultural competence limit utilization of services	4, 5, 8, 9, 10, 11, 12, 13, 14, 15, 17, 22, 24, 26	‐Cultural competence and language barriers ‐Mixed messages, missed opportunities	4, 9, 10, 11, 13, 15, 16, 17, 18, 19, 22, 26 5, 9, 11, 12, 14, 20, 18, 19, 21, 24	Describes how poor health literacy and misinformation prohibit health care access and/or appropriate care for those most in need. Nuances of literacy, language, ethnicity and race influence health care encounters for the marginalized where culturally responsive information and education must be implicit for successful health‐seeking within diverse communities
Motivation for treatment influences health service engagement	1, 2, 3, 6, 8, 7, 8, 10, 15, 17, 18, 19, 20, 21, 22, 24, 25, 26	‐Historical reluctance to seek treatment ‐Self‐efficacy and empowerment	1, 2, 3, 7, 8, 14, 17, 21, 22 1, 2, 4, 5, 6, 8, 12, 14, 16, 19, 21, 23, 24, 25	Describes narratives around health‐seeking where participants assess health service engagement within the context of their lives. Living environments, treatment setting, timing and readiness/preparedness for treatment uptake are considered. A historical reluctance to seek care due to previous negative experiences is investigated, in addition to characteristics of agency and empowerment amongst participants who were in a position to advocate for their health
Accessing services, a spectrum of experience—from discrimination to validation	1, 2, 3, 5, 6, 7, 8, 10, 11, 13, 17, 19, 20	‐Transitioning and co‐ordinated care	1, 2, 3, 6, 7, 8, 18	Outlines the barriers and facilitators to health service access. Describes impacts of racism and judgement that further compound barriers to access along with experiences of care and support, validating participant populations in their health‐seeking experience. Identifies the importance of co‐ordinated care models, particularly the alliance of health and social care, in aiding service access
Navigating a complex system in a complex society	2, 3, 6, 8, 13, 15, 16, 21, 24	‐Systemic obstacles to health care	2, 3, 4, 5, 6, 8, 10, 13, 15, 16, 18, 19, 20, 21, 24	Describes inherent obstacles to health care experienced by many participant populations and presents examples of thwarted opportunities for the marginalized in their health‐seeking. Systemic barriers, such as cost, convoluted referral processes, service availability, location and lack of transport infrastructure combine with social determinants to further disadvantage the marginalized as they attempt to engage with health services
Preferences for health care and expectations for systemic change	1, 2, 3, 4, 5, 6, 7, 8, 10, 11, 16, 17, 22, 24, 25, 26	‐Holistic and integrated care ‐Outreach and education	1, 2, 3, 4, 5, 7, 8, 10, 11, 19, 22 4, 5, 8, 11, 16, 17, 22, 24, 26	Outlines holistic and integrated health care—defined by participants as health care that addresses the ‘whole person’ including spiritual and psychological aspects as well as the physical body—as a preferred model of care for the marginalized. Participants expressed a desire for improved health education and the expectation that changing existing models of care to provide culturally appropriate services incorporating health promotion within communities, can have wide‐reaching and significant positive impacts amongst these populations

^a^ Corresponding first author ID and study details can be found in the data summary table (Table 3).

### 3.1 Understanding the patient within the context of family and community

Women who had little or no support from family or friends faced the most difficulties and, often, had worse linkages to health services.^20^

The role of the individual within the family and the impacts of support or lack thereof from family and community regarding health‐seeking was a pervasive narrative across each of the marginalized subgroups reported on in the reviewed literature (2, 8, 12, 13, 15–17, 19, 20, 22–25).

The literature illustrated that connection to family and community played a key role in motivating participant populations in their engagement with health services (12, 13, 23–25), though perceptions of connectedness to family and community could also perpetuate adverse health behaviours, such as problematic substance use (20, 23). Fears of exclusion from family and community or the shame that many participants reported in relation to aspects of health and health‐seeking were primary concerns for many participant populations (8, 12, 17, 20, 23, 25). A concern for ‘saving face’ was noted as a theme amongst some ethnic minority populations in our analysis (12, 17, 20). Seeking health care and information for stigmatized diseases, such as hepatitis C virus (HCV) (20, 21, 24, 25), HIV (18, 23), mental illness (16, 17), sexual and reproductive health problems (8, 11, 12) or for adverse health behaviours, such as intravenous drug use (1, 20, 21, 24, 25), caused anguish for participants due to perceptions of shame and stigma within their family and culture of origin. Additionally, a number of empirical papers reported that the notion of social support and subsequent feelings of inclusion and belonging appear to be important facilitators for participants to engage in a healthy lifestyle (1, 5, 13, 15, 16, 19, 22, 23, 26). When social support was lacking, study participants acknowledged the detrimental impacts on their health, as one participant in the study by Maneze et al. states: ‘To maintain health here will depend on our own resilience and resourcefulness because in the Philippines we have many family and friends who will invite us out and push us to be healthier (Julieta)’.^21^

### 3.2 Health and cultural beliefs influence health‐seeking

If they are ill, they will go to the fortune teller (thaˆ~y bo'i). Most I know go to Buddhist temple, the God things, voodoo people, fortune tellers in their language… If they get bad luck go to fortune teller, not doctor… If they are mentally ill, they go to fortune teller. (Lan Anh, 25‐year‐old female)^22^

Eighteen of the studies included in our synthesis are represented by this theme that encompasses participant narratives around the health impacts of risk‐taking, fatalism, gender and traditional medicines. Study authors reported the marked influence of cultural belief on an individuals' understanding of health, illness and treatment amongst the participant populations within this analysis (9, 12, 13, 15, 17, 20, 23) where the cause of the disease was often mentioned within a biopsychosocial context (13, 15) as exemplified by the following quote: ‘If I keep living in Australia, I may develop heart disease. I am alone here; no relatives; too much psychological pressure’.^23^

A prominent finding particularly amongst CALD populations in our analysis (15, 17–20, 22), showed preferences for culturally accepted health practices, such as prayer, spiritual practice and herbal medicine with authors noting that traditional medicines, remedies and notions of disease are often at odds with orthodox medical processes, diagnosis and treatment. The reviewed literature suggests the impacts of these health practices reinforce cultural identity and values while forging a sense of connection to an individual's country or culture of origin. For some marginalized groups, anecdotes of stoicism pervaded health beliefs where participants deemed that minimizing or tolerating suffering was culturally accepted (17, 20); enabled avoidance of medical treatment (22) or where health concerns may be superseded by meeting more pressing needs such as requirements for food and shelter (9).

#### 3.2.1 Perceptions of risk

I didn't worry about looking for a blood test or anything then, I didn't want to know if I did have it or not. I just thought I'd rather not know. (Ross)^24^

Cultural beliefs informed perceptions of health risk amongst some marginalized groups in the literature (5, 12, 13, 15, 18, 20, 23). Underrated and miscalculated risk behaviours pertaining to sexually transmitted diseases (11, 12, 18, 23) and contraction of blood‐borne viral infections, such as HIV and HCV, through intravenous drug use were reported in the reviewed literature (18, 20, 21, 23–25). Deeply rooted narratives of fatalism in relation to health behaviours, illness and risk were identified in some of the studies, exemplified in the following participant statement from Maneze et al. where study participants expressed simplified priorities in relation to life and death: ‘It's ok to die as long as I can eat good food. God will take care of it. If it is our time to die, we die, we cannot do anything about it (Carmen)’.^21^

#### 3.2.2 Cultural expectations related to gender and indications of female disadvantage

Some men exercised control over their female partners' use of healthcare services and refused to take them to a doctor, even when the women requested it.^25^

Authors reported the clear role of gender on the health attitudes of some participants, outlining instances where cultural beliefs and expectations relating to gender impacted access to health care, acceptance within family and community networks, social support and ultimately the well‐being of participant populations (12, 13, 18). Gender‐specific expectations signalled reduced opportunities and subsequent disadvantages in health‐seeking for women (8, 11–13, 17, 18). Eight of the studies included in our analysis report on women from CALD or refugee backgrounds and their disadvantage in accessing health services (8, 10–13, 15, 18, 19). These participants commonly expressed difficulties reconciling the reality of cultural expectations and acculturation in a new society. The ramifications of cultural shaming and its particular pertinence to women are clear in the following quote from McMichael and Giffords' 2010 research reporting on the sexual health literacy of refugee youth:

I think they would shame her…. They will talk about it a lot, a lot…. She can't face anyone in our culture because she make shame on it. (Basima, female, Iraq, 16 years, FGD)^26^

### 3.3 Lack of information and poor cultural competence limit utilization of services

I don't know what diabetes is. All the doctors say I have diabetes, but no one has explained what diabetes is. Sometimes I feel like asking them this question because some people say diabetes is a big thing.^27^

The literature included in this review identified significant gaps in health literacy (4, 5, 8–15, 17–22, 24, 26) across all marginalized subgroups studied. In our analysis, this theme represents findings related to inadequate knowledge pertaining to a health condition or disease prevention; risk factors and cause of disease; diagnostic procedures or treatment. Additionally, it refers to participants lacking information regarding types of available services and roles of specialists (3, 7, 8).

#### 3.3.1 Cultural competence & language barriers

Complicated explanations and excessive jargon created feelings of disengagement and discontent.^28^

Consideration of culturally responsive care was identified as lacking in a number of studies included in our analysis (13, 15, 16, 19, 22) where participants reported discriminatory attitudes by staff (22) or interpreters communicating in the wrong dialect (9). In their study, Kendall and Barnett (2015) observed that inadequate cultural sensitivity and poor communication alienated indigenous Australians in their health‐seeking with the use of colonial communication styles and medical jargon (22).^28^ A number of studies (4, 22, 26) report a need for culturally appropriate communication for the purposes of relating health information to indigenous Australians, focused on storytelling and yarning as best practice methods to engage these populations. In our analysis, language barriers (4, 9–11, 13, 15–17, 18–22) were noted where various participant populations reported difficulty communicating with health providers; accessing services due to a lack of proficiency in English or where medical jargon prohibited inclusion and shared understandings of health as expressed in this comment from an Aboriginal participant:

They just gave me information to go off and read it myself. … So no one actually talked, all they gave me was a kit to take home with me. … I haven't really read it, no, I get more from you know, sitting down and speaking to someone, verbally and stuff like that.^29^

The notion of cultural responsiveness in health care provision extends to the misunderstanding and invalidation of LGBTQI+ and gender diverse people, described by two of the study cohorts representing disadvantaged youth populations (5, 8). Analysis of the reviewed literature indicates that provider attitudes in health care provision remain governed by predominately heteronormative attitudes. Participants from these studies highlight a lack of gender diverse cultural understanding and inclusivity amongst staff and within service structures, that is exemplified by the following participant quote: “ ‘I said to her [school counsellor] are there any support groups or anything that I can go to, anyone I can talk to', and all she said to me was, ‘Just wait until you're at uni. It will be fine then. You'll meet lots of queer people there’.”^30^

#### 3.3.2 Mixed messages, missed opportunities

I go to ask this doctor, ‘I hear [from] someone, treatment’. He say ‘What stupid western, where you listen from? Where you hear this stupid story?’ He say, ‘No way. Got no treatment! You got that all your life’. (Van, 34‐year‐old Vietnamese Australian male)^31^

The reviewed literature shows that confused messaging resulted in missed opportunities for timely treatment (21), convoluted referral processes (21), hidden and prohibitive costs (8, 21) and forgone care (5) for the marginalized and socioeconomically disadvantaged. Numerous participant groups reported on in the included studies claimed incorrect knowledge about aspects of health prevention, illness and disease leading to confusion and misunderstanding (9, 11, 14, 18, 19, 21, 24). Misinformation concerning treatment for some diseases, such as diabetes, sexual and reproductive health (11, 12, 24) or blood‐borne viruses, such as HCV or HIV was also reported in the literature (11, 12, 18, 21, 24). In some instances, the research identified the source of information from health care providers was profoundly lacking (5, 14, 18, 24) or misinformed (18, 21) while clear needs for improved communication from physicians—relating diagnoses and disease management to patients—were highlighted by study authors from our analysis (7, 14, 15, 18, 24).

### 3.4 Motivation for treatment influences health service engagement

You don't want to waste your chance and stuff it up. Because at the moment, to be truthful, I'm not ready to stop using yet. (Noi, 28‐year‐old Lao‐Australian male)^31^

Perceived success in health‐seeking amongst the populations represented within this review was related to opportunities that arose in particular contexts. Examples include instances when the availability of care was found beyond the scope of ‘normal’ operating hours and service provision (1, 2, 6, 8) and where the likelihood of receiving the care was determined by participant populations and study authors as being in the right place at the right time (1, 2, 6, 10, 20, 21, 24). Missed opportunities to access health care were determined by both the patient's physical environment (1, 2, 20) and the context within which care or treatment was being offered, accessed or overlooked (1, 2, 18). For individuals with unstable accommodation, there was difficulty in committing to treatment or appointment schedules (7, 21). Environment and its impact on risk behaviours and treatment‐seeking are highlighted in some studies and of note in this analysis (1, 2, 20, 24). Abbot et al. (2016, 2017) observed missed opportunities for women transitioning between prison and community care, where receiving health care was elusive for those who were not in the prison system long enough or conversely, instances where for others prison was the most reliable place to receive health care (1, 2). Similarly, Treloar et al. noted amongst their indigenous participant population that receiving treatment for HCV within the prison was reported as convenient and supportive for some and a deterrent for others who felt that side effects of treatment would render them further vulnerable within an institutionalized environment (24).

Motivation for treatment of substance abuse was reported with enthusiasm amongst some participants (21) but was approached with equal measures of wariness and pragmatism by individuals who were concerned about side effects, being ready to quit and getting the timing of subsidized treatment right (20, 21, 24). Amongst Filipino, Thai and Chinese Australians there was a reluctance to seek help for mental health concerns (15, 17, 19). Across the studies included in this synthesis, reports of treatment motivation and patient satisfaction were higher for services that were founded/developed for specific populations, such as refugee and asylum seekers (10) and Aboriginal Community‐controlled Health Organisations (3, 22, 25, 26).

#### 3.4.1 Historical reluctance to seek treatment

The moment I tell them I'm of Aboriginal descent it goes down a notch and they speak to me like I'm not worth talking to or I'm too stupid to know. (Murri, community elder, city, group interview)^28^

Previous negative experiences in seeking health care were reported in the reviewed literature as a deterrent to continued health‐seeking, impacting continuity of care (1–3, 7) and perpetuating mistrust in a medical model built on a hierarchical power imbalance (3, 8, 14, 22). In the case of indigenous Australians, Kendall et al. have highlighted the cultural chasm in delivering medicine ‘historically associated with colonization, categorization and separation of families’ to first nations peoples (22).^28^ Mistrust of the medical system precipitated a reluctance to seek timely treatment posing increased risks to health (2, 3, 8, 17, 22) as the following quote suggests: ‘When they don't help you enough, you sick of it. You already know what's gonna happen, [how] it's gonna turn out. They not gonna help you properly. You know yourself. You been there too many times’ (Pack, 29‐year‐old Lao‐Australian male).^31^

#### 3.4.2 Self‐efficacy and empowerment

I always went on the fact that as long as I look after my liver I will be alright, so that was when I went nil alcohol or anything to make sure my liver was strong—I sort of took the treatment on myself.^29^

Degrees of self‐efficacy and empowerment pertaining to health‐seeking were reported across 12 of the included studies (1, 2, 4–6, 8, 12, 14, 16, 19, 23–25). Notable within this theme are narratives of empowerment to disengage from services or processes (5, 6), self‐advocacy within the patient/doctor dynamic (14) and consumer rights to give feedback and lodge complaints (4). Study authors reported empowerment of participant populations after positive experiences of support through engagement with health workers or services (1, 2, 8, 14, 16). The reported empowerment of refugee youth (12) and indigenous peoples (23–25) who asserted locus of control over health behaviours and management of the disease is significant.

### 3.5 Accessing services—A spectrum of experience, from discrimination to validation

…so whenever they saw that we are wearing the scarf…they judge [us]. (Female, 18 years, refugee)^32^

Stories of health service access amongst our study populations were marked by opposing themes of discrimination (1, 2, 3, 5–8, 13, 17, 20) and validation (6, 7, 10). Study authors reported themes of ‘feeling like I mattered’ (7) and feeling heard, that validated study participants' rights to access services. Equally, perceived judgement, discrimination, racism and the use of ‘labelling’ (5, 7, 17) to dismiss the concerns of health‐seeking individuals (as described by participants), reinforced barriers to health care access and contributed to forgone care, perpetuating and entrenching marginalization. In our analysis study, authors identified stigma as a barrier to accessing health services and treatment for marginalized populations with chronic illness, low socioeconomic status, culturally diverse backgrounds, histories of incarceration or problematic substance use (1, 2, 8, 16, 24). As a result of the participant reported discrimination faced by the range of subgroups within our marginalized study populations (1–3, 5–8, 13, 20, 21) we identified that attitudes of staff and health care providers towards marginalized persons attempting access to services was significant and influential across this review cohort. Additionally, authors observed that successfully challenging entrenched stigma and forging legitimate claims to care were often seen as turning points in experiences of health care access (1, 2, 14, 20). In our analysis, the use of technology, such as the internet and social media, was recognized as an aid to health service access (5, 8, 11, 19): ‘I like when booking and that kind of stuff is online… talking on the phone's very alien to me… I prefer not to. So, I like to be able to manage appointments and all that kind of stuff online’ (Female, 23 years, sexuality diverse, homeless).^32^

#### 3.5.1 Transitioning and co‐ordinated care

I was always able to access them no matter what. So that was like a one stable place I had from fourteen all the way through to, I was about seventeen.^33^

This theme reports experiences of access to care co‐ordination and interservice referrals to manage health. The potential importance of the roles of care coordinators, mentors and various providers across health and social services, and the degree to which these elements interacted, had reportedly direct and often profound impacts on the individuals represented within these studies (1–3, 6–8). The importance of health care co‐ordination described by research participants and authors was highlighted amongst marginalized youth when transitioning from paediatric to adult care (8) or from homelessness to emergency housing (6, 7); for individuals of low socioeconomic status attempting to manage chronic illnesses such as diabetes (3); and for stigmatized ex‐prison populations seeking community‐based health care postrelease (1, 2).

A number of participants identified defined yet small ‘windows of opportunity’ where the receipt of timely and co‐ordinated care influenced their health journeys (1, 2, 6, 8), as shown by the following participant quote: ‘A plan, an appointment, set for a week after you're out … I think if you make that appointment quickly, you've got that little bit of time where you can get them in’ (Participant 35).^34^ Conversely, when these windows of opportunity were not supported by interservice communication, follow‐up or referral, researchers observed the impacts on the health seeker could be devastating (6–8, 18) detailed by this participant experience:

When I start feeling sick like the flu I went to my doctor. After that he said to me ‘When you say the symptoms’ he say, ‘Could be that (HIV)… But I don't think so because you are a lady’… And later on, when he discovered it he said ‘I don't know who the gentleman is and I don't want to know’. (Argentinean woman, diagnosed 1996)^25^

### 3.6 Navigating a complex system in a complex society

Finding services has been difficult, remembering to get referrals and then to follow up on them, finding the time to do those things… I do know it's something I should into because it bothers me, frustrates me a lot… I always tend to lose touch with my mental health professionals right when I need them… The times I could really use someone is when it's hard to get access. (Female, 23 years, sexuality diverse, homeless)^32^

Negotiating the health care system was described as confusing, intimidating and overwhelming by many of the participant populations included in this review (3, 8, 13, 15, 16, 21). Accessing services, lack of or under‐resourced services and inadequate knowledge about how to locate and engage with appropriate services were reported by study authors as typical difficulties that challenged marginalized populations navigating the health system (2, 3, 6, 8, 13, 15, 16, 24).

#### 3.6.1 Systemic obstacles to health care

I'm not sure whether it's because of our area or whether it's the fact, you know, that I'm a Medicare patient, I'm not sure, but I do feel that uh…there's not really enough information about what services are actually available to you, and if…if they are available, well, you seem to have to fight tooth and nail to get them, and I do feel that that's quite unfair. (Alison; 50s; tablets; diagnosed 8 years)^35^

For the marginalized populations in our analysis, systemic obstacles, such as eligibility criteria (6, 18, 21); lack of service integration (4, 6); referral processes (2, 21); discrimination (3, 5, 21); negotiating welfare, disability or child support (2, 4, 21); protracted waiting times (2, 20, 21, 24) and cost (3, 13, 15, 21)—often prohibitive and hidden^32^—were compounded by social and structural determinants such as education and health literacy (8, 24); age and gender/identity (5, 6, 8, 13); cultural background (13, 18, 20, 21, 24); transport availability (3, 4); housing/accommodation (3, 21) and available or accessible services.

Despite the overarching theme of complexity and difficulty in navigating the Australian health system described by authors of many of the studies, three participant populations from CALD backgrounds reported favourably on their experiences of health system navigation compared to their country of origin (10, 15, 19). As the following participant contributing to research on a refugee and asylum seeker health service noted: ‘…we never had a good government or a good system, and so we have never been treated well. And compared to that, coming here where we are treated well and we have all the services around me—so I just value everything here’ (Male, 33 years old, Afghanistan).^36^

Participants placed value on support and information provided to aid them in their navigation of a fragmented system (6, 8), a lack of which typically hindered their health journeys (2, 6, 8, 13, 16, 24). Some expressed a need for greater education and information about health system processes and pathways, clearly defined by the following statement: ‘A support person to help them go through the process of accessing—just to give them some guidance… and maybe just checking up on them every now and then to… see if that's going well. If it's not, they can help guide them on the right way to do that’ (Male, 18 years, homeless).^32^

### 3.7 Preferences for health care and expectations for systemic change

Welcoming signals—such as rainbow and Aboriginal flags—were seen as positive ways for services to recognise diversity.^32^

Born out of perceived negative experiences with health services, participant populations across the range of marginalized subgroups from this review outlined preferences for what constitutes acceptable and engaging health services (2–8, 10, 11, 16, 17, 22, 24–26). Friendly and competent staff working within flexible service structures where interservice communication was embraced were valued (6, 8). Inclusivity has been identified as a valued attribute across the diverse participant population base within this review and incorporates the inclusion of stigmatized ex‐prison populations (1, 2) or those with histories of substance misuse (2); populations with lower English literacy (4); sexual minority groups (5, 8) and for Australian first nations peoples (2, 3, 8, 24–26) who reported favourably on experiences with Aboriginal‐specific health services.

#### 3.7.1 Holistic and integrated care

A place that provides medical help and accommodation at the same time. (Interview 10)^37^

The groups within this review reported a desire for holistic and integrated services. Participant groups outline such services as health care that considers all aspects of an individuals' physical and psychological needs in addition to their social needs. Integrated services are defined as co‐ordinated primary and allied health services that work together, can be accessed close to each other or under the same roof. Study participants commonly viewed their well‐being as multidimensional and thus placed value on and idealized the importance of integrated care models that incorporated elements of allied health (1, 2, 3, 4, 7, 10), complementary or traditional medicines (2, 22) and social care (4, 7).

#### 3.7.2 Outreach and education

Providing mental health training to interpreters could have prevented lots of problems. (Arabic‐speaking consumer)^38^

Nine of the studies included in this review outlined the importance of community‐based outreach, education and health promotion (4, 5, 8, 11, 16, 17, 26). Requests from participants for educating families, spiritual leaders (16, 17, 19) and whole communities about mental illness and sexual and reproductive health were perceived as instrumental to support marginalized health seekers from refugee and CALD backgrounds (11, 16, 17) as well as sexual minority youth populations (5). Marginalized youth and indigenous health seekers mentioned the need for improved explanations and education concerning diagnoses, disease processes and prevention from health care providers (8, 22, 24).

## 4 DISCUSSION

This review identifies several commonalities in the experience of health services amongst marginalized populations in Australia. Australia's diverse sociocultural landscape suggests the experiences of health‐seeking for marginalized people is not easily defined and there is an imperative to acknowledge the unique characteristics and requirements of differing groups, such as indigenous peoples, asylum seekers and marginalized youth.^2^, ^9^ However, despite clear nuances of health service experience amongst each of the marginalized subgroups within this analysis, the power of appraising the collective experience, and indeed the goal of synthesis,^13^ tells a bigger story of systemic failure and multiple marginalization. Narratives of belonging are encapsulated by themes of family, community, culture and belief that inform experiences of navigation and learning, bound by a common vision for a health system that can better meet complex needs. Similar themes pertaining to health‐seeking amongst CALD communities are reported internationally^19^ and considerable barriers to equitable health care access for the disadvantaged and marginalized have been acknowledged in the research literature.^2^, ^6^ Amongst the Australian marginalized populations in our study, we found that these barriers exist on and across individual, family, community, service provision and systemic levels and are influenced by both social and structural determinants of health. This finding highlights that barriers to health care in contemporary Australia are multidimensional and often entrenched at systemic levels.^15^ The result of these systemic barriers is a common denominator of disadvantage distinct from individual contexts or backgrounds, that pervades many marginalized populations attempting access to health services.

Our review has outlined significant gaps in health literacy amongst the various participant populations while highlighting a general lack of culturally responsive care from service providers. This finding is supported by research that indicates culturally responsive communication and care is essential for equitable health care delivery (Clinical excellence commission, 2011) but is commonly lacking in the realities of clinical practice.^8^ Experiences of suboptimal health care and failed attempts to access appropriate care due to poor communication and cultural insensitivity, were commonly reported across the studies included in this review. Previous research suggests the outcomes of ineffective communication in health settings lead to misunderstanding, inappropriate interventions, negligence and forgone care.^2^, ^8^, ^39^ We note that once marginalized individuals are engaged within the health system, health literacy amongst this population is proportionate to clinician competence in patient education. For this reason, effective delivery of culturally responsive care is reliant on clinician competence and effective communication skills. Culturally responsive communication from providers leads to increased access to health services and improved health outcomes for marginalized populations.^8^ Guidelines for training health workers and staff in cultural responsiveness while supporting providers and systems that value and encourage these skills require further development. Championing reforms to mandate the provision of culturally responsive communication in all aspects of health care can ensure that marginalized populations are supported with the correct information to make informed choices, empowering them in their health‐seeking and reducing disadvantage.^40^

This review has found that people who are marginalized within Australian communities express a need for integrated care models. Integration across systems of medicine to include Traditional and Complementary Medicines is a part of the WHO's traditional medicine strategy to promote universal health coverage.^41^ This strategic push is interesting in light of our analysis, which revealed that modes of CM, such as herbal medicine, were preferred by some of the CALD participant populations. Emerging evidence suggests that integration across systems of medicine to incorporate CM additional to an array of medical and allied health services may be valuable in addressing health delivery for the marginalized.^42^ Furthermore, our analysis suggests that an integrated approach to health and social care may help marginalized populations in their navigation of a complex system. Consistent and compelling evidence that health is shaped by social determinants^4^ means that aiding marginalized people in their health‐seeking commonly requires coordinated input from a range of services. In community settings, social services can be a vital element in facilitating access to health care for the marginalized.^6^ Social care has been defined as ‘services that address health‐related social risk factors and social needs’.^4^ Multiple factors, such as homelessness, illiteracy, poverty, accessibility and transport issues, additional to needs specific to individuals of diverse sociocultural backgrounds, can potentially inhibit the marginalized from successfully engaging with health services. Research from the United States identifies one of the three key imperatives for effective integration is an appropriately trained workforce. Further recommendations that educational institutions for all health professions mandate competency‐based training in social care and expanded curricula for learning about social determinants of health have also been outlined.^4^ These recommendations can be applied within an Australian teaching and health service landscape to help address the inequalities faced by those populations marginalized by existing systems.

### 4.1 Limitations

Our review may have been limited by the exclusion of quantitative analyses. However, due to the broad scope of the review question and the subsequent number of papers identified through the systematic search process, our focus on qualitative studies was the most appropriate method to meaningfully answer the research question. We only included published literature and the perspectives represented in our review are limited to metropolitan dwelling Australian marginalized populations. There are significant and important particularities unique to health‐seeking for populations located in rural and remote Australia.^7^ This suggests that geographical location is itself a key social determinant that has a significant impact on the health service experience of these populations, justifying our recommendation that a future examination of the literature specific to rural and remote dwelling groups is warranted. Researcher reflexivity is an essential component of meta‐ethnographic reporting.^14^ As such the review team acknowledge the influence of a researcher's individual worldview and disclose their respective backgrounds in health research (J. A., A. S., K. B.), sociology (J. A.) and clinical roles in allied health within PHC settings (K. B.).

## 5 CONCLUSION

Marginalized populations in Australia experience inequalities and barriers to health care across micro, meso and macro levels of system navigation. There is a need to improve health literacy and support the provision of culturally responsive communication, and these changes need to be addressed as interdependent elements within health systems if health inequalities are to be tackled. Furthermore, integrated health and social care may be central to meeting the myriad and complex needs of marginalized and disadvantaged groups. The populations represented by this analysis are diverse, though their collective experiences provide a compelling narrative that unites them in their health‐seeking.

## CONFLICT OF INTERESTS

The authors declare that there are no conflict of interests.

## Supporting information

Supporting information.

## Data Availability

The data that support the findings of this study are available from the corresponding author upon reasonable request.

## References

[1] Social Determinants of Health. World Health Organisation. 2021. Accessed November 9, 2021. https://www.who.int/teams/social-determinants-of-health

[2] Johnstone M‐J , Kanitsaki O . Health Care Provider and Consumer Understandings of Cultural Safety and Cultural Competency in Health Care: An Australian Study. RMIT University; 2007. 19175250

[3] Luchenski S , Maguire N , Aldridge RW , et al. What works in inclusion health: overview of effective interventions for marginalised and excluded populations. Lancet. 2018;391(10117):266‐280. 29137868 10.1016/S0140-6736(17)31959-1

[4] National Academies of Sciences Engineering, Medicine. *Integrating Social Care into the Delivery of Health Care: Moving Upstream to Improve the Nation's Health*. 2019. 31940159

[5] Cheraghi‐Sohi S , Panagioti M , Daker‐White G , et al. Patient safety in marginalised groups: a narrative scoping review. Int J Equity Health. 2020;19(1):1‐26. 10.1186/s12939-019-1103-2 PMC7014732 32050976

[6] Goeman D , Howard J , Ogrin R . Implementation and refinement of a community health nurse model of support for people experiencing homelessness in Australia: a collaborative approach. BMJ Open. 2019;9(11):e030982. 10.1136/bmjopen-2019-030982 PMC6887079 31748297

[7] Australian Institute of Health and Welfare Australia's health 2020: In Brief. 2020. Accessed November 9, 2021. https://www.aihw.gov.au/reports/australias-health/australias-health-2020-in-brief/contents/summary

[8] Minnican C , O'Toole G . Exploring the incidence of culturally responsive communication in Australian healthcare: the first rapid review on this concept. BMC Health Serv Res. 2020;20(1):1‐14. 10.1186/s12913-019-4859-6 PMC6947994 31910837

[9] Echeverri M , Chen AM . Educational interventions for culturally competent healthcare: developing a protocol to conduct a systematic review of the rationale, content, teaching methods, and measures of effectiveness. J Best Pract Health Prof Divers. 2016;9(1):1160‐1177. 34296221 PMC8294596

[10] Dawson A , Jackson D . The primary health care service experiences and needs of homeless youth: a narrative synthesis of current evidence. Contemp Nurse. 2013;44(1):62‐75. 23721389 10.5172/conu.2013.44.1.62

[11] Jones B , Heslop D , Harrison R . Seldom heard voices: a meta‐narrative systematic review of Aboriginal and Torres Strait Islander peoples healthcare experiences. Int J Equity Health. 2020;19(1):1‐11. 10.1186/s12939-020-01334-w PMC7734845 33317556

[12] France EF , Cunningham M , Ring N , et al. Improving reporting of meta‐ethnography: the eMERGe reporting guidance. BMC Med Res Methodol. 2019;19(1):1‐13. 30709371 10.1186/s12874-018-0600-0 PMC6359764

[13] Noblit GW , Hare RD . Meta‐ethnography: Synthesizing Qualitative Studies. Vol 11. Sage; 1988.

[14] Sattar R , Lawton R , Panagioti M , Johnson J . Meta‐ethnography in healthcare research: a guide to using a meta‐ethnographic approach for literature synthesis. BMC Health Serv Res. 2021;21(1):1‐13. 33419430 10.1186/s12913-020-06049-w PMC7796630

[15] Cruwys T , Berry H , Cassells R , et al. Marginalised Australians: characteristics and predictors of exit over ten years 2001‐10. University of Canberra, Australia; 2013.

[16] Critical appraisal skills programme. CASP qualitative checklist, 2018. Accessed November 9, 2021. https://casp-uk.net/casp-tools-checklists/

[17] Long HA , French DP , Brooks JM . Optimising the value of tc(CASP) tool for quality appraisal in qualitative evidence synthesis. Res Methods Med Health Sci. 2020;1(1):31‐42.

[18] Campbell R , Pound P , Pope C , et al. Evaluating meta‐ethnography: a synthesis of qualitative research on lay experiences of diabetes and diabetes care. Soc Sci Med. 2003;56(4):671‐684. 12560003 10.1016/s0277-9536(02)00064-3

[19] Garrett CR , Gask LL , Hays R , et al. Accessing primary health care: a meta‐ethnography of the experiences of British South Asian patients with diabetes, coronary heart disease or a mental health problem. Chronic Illn. 2012;8(2):135‐155. 22414446 10.1177/1742395312441631

[20] Abbott P , Magin P , Davison J , Hu W . Medical homelessness and candidacy: women transiting between prison and community health care. Int J Equity Health. 2017;16:1‐10. 28728555 10.1186/s12939-017-0627-6 PMC5520372

[21] Maneze D , Ramjan L , DiGiacomo M , Everett B , Davidson PM , Salamonson Y . Negotiating health and chronic illness in Filipino‐Australians: a qualitative study with implications for health promotion. Ethn Health. 2018;23(6):611‐628. 28271720 10.1080/13557858.2017.1294656

[22] Ho HT , Maher L . Co vay co tr[image omitted] (What goes around comes around): culture, risk and vulnerability to blood‐borne viruses among ethnic Vietnamese injecting drug users. Drug Alcohol Rev. 2008;27(4):420‐428. 18584393 10.1080/09595230801914743

[23] Gholizadeh L , DiGiacomo M , Salamonson Y , Davidson PM . Stressors influencing Middle Eastern women's perceptions of the risk of cardiovascular disease: a focus group study. Health Care Women Int. 2011;32(8):723‐745. 21767097 10.1080/07399332.2011.562999

[24] Thompson SC , Bonar M , Greville H , et al. ‘Slowed right down’: insights into the use of alcohol from research with Aboriginal Australians living with HIV. Int J Drug Policy. 2009;20(2):101‐110. 18400484 10.1016/j.drugpo.2008.02.003

[25] Körner H . Late HIV diagnosis of people from culturally and linguistically diverse backgrounds in Sydney: the role of culture and community. AIDS Care. 2007;19(2):168‐178. 17364395 10.1080/09540120600944692

[26] McMichael C , Gifford S . Narratives of sexual health risk and protection amongst young people from refugee backgrounds in Melbourne, Australia. Cult Health Sex. 2010;12(3):263‐277. 19904650 10.1080/13691050903359265

[27] Jiwrajka M , Mahmoud A , Uppal M . A Rohingya refugee's journey in Australia and the barriers to accessing healthcare. BMJ Case Rep. 2017;2017:bcr-2017-219674. 10.1136/bcr-2017-219674 PMC5612524 28487305

[28] Kendall E , Barnett L . Principles for the development of Aboriginal health interventions: culturally appropriate methods through systemic empathy. Ethn Health. 2015;20(5):437‐452. 24993550 10.1080/13557858.2014.921897

[29] Treloar C , Jackson C , Gray R , et al. Care and treatment of hepatitis C among Aboriginal people in New South Wales, Australia: implications for the implementation of new treatments. Ethn Health. 2016;21(1):39‐57. 25665723 10.1080/13557858.2015.1004870

[30] Pallotta‐Chiarolli M , Martin E . Which sexuality? Which service: bisexual young people's experiences with youth, queer and mental health services in Australia. J LGBT Youth. 2009;6(2‐3):199‐222.

[31] Coupland H , Day C , Levy MT , Maher L . Promoting equitable access to hepatitis C treatment for Indo‐Chinese injecting drug users. Health Promot J Austr. 2009;20(3):234‐240. 19951245 10.1071/he09234

[32] Robards F , Kang M , Steinbeck K , et al. Health care equity and access for marginalised young people: a longitudinal qualitative study exploring health system navigation in Australia. Int J Equity Health. 2019;18:41. 30832651 10.1186/s12939-019-0941-2 PMC6399978

[33] Black EB , Fedyszyn IE , Mildred H , et al. Homeless youth: barriers and facilitators for service referrals. Eval Program Plann. 2018;68:7‐12. 29454263 10.1016/j.evalprogplan.2018.02.009

[34] Abbott P , Davison J , Magin P , Hu W , Magin PJ . ‘If they're your doctor, they should care about you’: women on release from prison and general practitioners. Aust Fam Physician. 2016;45(10):728‐732. 27695723

[35] Cuesta‐Briand B , Saggers S , McManus A . “It still leaves me sixty dollars out of pocket”: experiences of diabetes medical care among low‐income earners in Perth. Aust J Prim Health. 2014;20(2):143‐150. 23448698 10.1071/PY12096

[36] McBride J , Block A , Russo A . An integrated healthcare service for asylum seekers and refugees in the South‐Eastern Region of Melbourne: Monash Health Refugee Health and Wellbeing. Aust J Prim Health. 2017;23(4):323‐328. 28756817 10.1071/PY16092

[37] Darbyshire P , Muir‐Cochrane E , Fereday J , Jureidini J , Drummond A . Engagement with health and social care services: perceptions of homeless young people with mental health problems. Health Soc Care Community. 2006;14(6):553‐562. 17059497 10.1111/j.1365-2524.2006.00643.x

[38] Prasad‐Ildes R , Ramirez E . What CALD consumers say about mental illness prevention. *Aus e‐J Adv Men Heal*. 2006;5(2):126‐131.

[39] O'Toole G . Communication‐eBook: Core Interpersonal Skills for Healthcare Professionals. Elsevier Health Sciences; 2020.

[40] Gill G , Babacan H . Developing a cultural responsiveness framework in healthcare systems: an Australian example. Divers Health Care. 2012;9(1):45‐55.

[41] World Health Organisation . WHO Global Report on Traditional and Complementary Medicine 2019. 2019.

[42] Baker K , McDonald J , Steel A . Tackling health inequity: a commentary on the potential of acupuncture to improve health outcomes of marginalised populations. Acupunct Med. 2020;39:553‐537. 33280395 10.1177/0964528420961404

