# Supplementary material for: Experiences, perceptions and expectations of health services amongst marginalized populations in urban Australia: A meta‐ethnographic review of the literature
Source: Health Expect. 2021 Dec 14;25(5):2166–87. doi: 10.1111/hex.13386 (PMC9615052; doi:10.1111/hex.13386)
Supplement: Supplementary file 1 — Supporting information. [file HEX-25--s001.docx]

|  | Embase (OVID) search string terms with results |
| --- | --- |
| 1 | (marginalised or vulnerable or disadvantaged or at risk or minority or underserved).ti.ab. (440020) |
| 2 | vulnerable population/ (16085) |
| 3 | health behavior/ (64861) |
| 4 | (experience* or Perception*).mp. or Preference*.ti.ab (1965063) |
| 5 | health knowledge, attitudes, practice.mp. or attitude to health/ (109872) |
| 6 | patient preference/ (17736) |
| 7 | patient satisfaction/ (134982) |
| 8 | patient acceptance of health care.mp. (252) |
| 9 | exp health service/ (5272102) |
| 10 | health care utilization/ or health care/ (179923) |
| 11 | health provision.mp. (497) |
| 12 | Australia/ 151524 |
| 13 | Australia.ti.ab. (116916) |
| 14 | 1 or 2 |
| 15 | (communit* or group* or population*)ti.ab. (7088001) |
| 16 | 14 and 15 (224555) |
| 17 | 9 or 10 or 11 (5291359) |
| 18 | 3 or 4 or 5 or 6 or 7 or 8 (2192969) |
| 19 | 13 and 16 and 17 and 18 (535) |
| 20 | From 19 keep 1-535 |

*search conducted 05/03/2020

|  | Psych Info search string terms with results |
| --- | --- |
| 1 | Health knowledge, attitudes, practice (25218) |
| 2 | Attitude to health (91473) |
| 3 | Patient satisfaction (15701) |
| 4 | Patient preference (5390) |
| 5 | Patient acceptance of health care (11050) |
| 6  7  8 | Health behaviour (77340)  Experience*.ti.ab. OR perception*.ti.ab. OR preference*.ti.ab. (0)  Experience*.ti.ab. (0) |
| 9 | AB experience* OR ABperception* OR AB preference (864,971) |
| 10 | Vulnerable populations (7753) |
| 11 | Marginalised OR vulnerable OR disadvantaged OR minority OR underserved OR at risk (503,193) |
| 12 | Communit* OR group* OR population (1,540,784) |
| 13 | MA health services (56,346) |
| 14 | AB Australia (26,559) |
| 15 | S11 OR S12 (509,193) |
| 16 | S13 AND S16 (284,003) |
| 17 | S1 OR S2 OR S3 OR S5 OR S6 OR S10 (986,895) |
| 18 | S13 AND S14 AND S16 AND S17 |

*search conducted 01/03/2020

|  | Informit search string terms with results |
| --- | --- |
| 1 | Health knowledge, attitudes, practice OR attitude to health OR patient satisfaction OR patient preference OR patient acceptance of health care (773) |
| 2 | (health behaviour) OR experience* OR perception * OR preference* (8,324) |
| 3 | Vulnerable populations (53) |
| 4 | Marginalised OR disadvantaged OR underserved OR minority OR (at risk) (2,993) |
| 5 | Population* OR communit* OR group* (18,820) |
| 6 | (health services) OR (health care) OR (health care utilisation) OR (health provision) (18,364) |
| 7 | Australia (37,099) |
| 8 | (population* OR communit* OR group*) AND (marginalised OR disadvantaged OR underserved OR minority OR (at risk)) (1,498) |
| 9 | ((health behaviour) OR experience* OR perception* OR preference*) OR ((health knowledge, attitudes, practice) OR (attitude to health) OR (patient satisfaction) OR (patient preference) (8,791) |
| 10 | ((population* OR communit* OR group*) AND (marginalsied OR disadvantaged OR underserved OR minority OR (at risk)) OR (vulnerable populations) (1,535) |
| 11 | ((population* OR communit* OR group*) AND (marginalised OR disadvantaged OR underserved OR minority OR (at risk)) OR (vulnerable populations) AND (health behaviour) OR experience* OR perception* (112) |

*search conducted 09/03/2020

|  | Medline (OVID) serach string terms with results |
| --- | --- |
| 1 | Health behavior (48847) |
| 2 | (experience* Perception*).mo. or Preference*.ti.ab. (1532118) |
| 3 | ((marginalised or vulnerable or at risk or underserved or disadvantaged or minority) adj3 (population* or communit* or group*)).ti.ab. |
| 4 | (experience* or Perception*).mp. or Preference*.ti.ab. (1532118) |
| 5 | Health Knowledge, Attitudes, Practice/ (108552) |
| 6 | Attitude to Health (82770) |
| 7 | Patient Satisfaction/ (80235) |
| 8 | Patient Preference/ (8042) |
| 9 | “Patient Acceptance of Helath Care”/ (45238) |
| 10 | Exp Health Services/ (2094699) |
| 11 | Health care utilisation.mp. (581) |
| 12 | Health care.mp. (774999) |
| 13 | Health provision.mp. (390) |
| 14 | Australia.ti,ab. (90228) |
| 15 | 10 or 11or 12 or 13 (2572487) |
| 16 | 1 or 2 or 5 or 6 or 7 or 8 or 9 (1774591) |
| 17 | 3 and 14 and 15 and 16 (178) |
| 18 | Australia/ (100780) |
| 19 | 3 and 15 and 16 and 18 (142) |
| 20 | Vulnerable Populations/ (10049) |
| 21 | 3 or 20 (61690) |
| 22 | 14 or 16 (153442) |
| 23 | 15 and 16 and 21 and 22 (287) |

*search conducted 06/03/2020
